# Supplementary material for: Discovery and Characterization of Two Selective Inhibitors for a Mu-Class Glutathione S-Transferase of 25 kDa from Taenia solium Using Computational and Bioinformatics Tools
Source: Biomolecules. 2024 Dec 25;15(1):7. doi: 10.3390/biom15010007 (PMC11760891; doi:10.3390/biom15010007)
Supplement: Supplementary file 1 [file biomolecules-15-00007-s001.zip › biomolecules-3340093-supplementary.pdf]

biomolecules

MDPI

Supplementary Materials:

2D interaction diagram between GSH and Ts25GST in the pre-MD model.

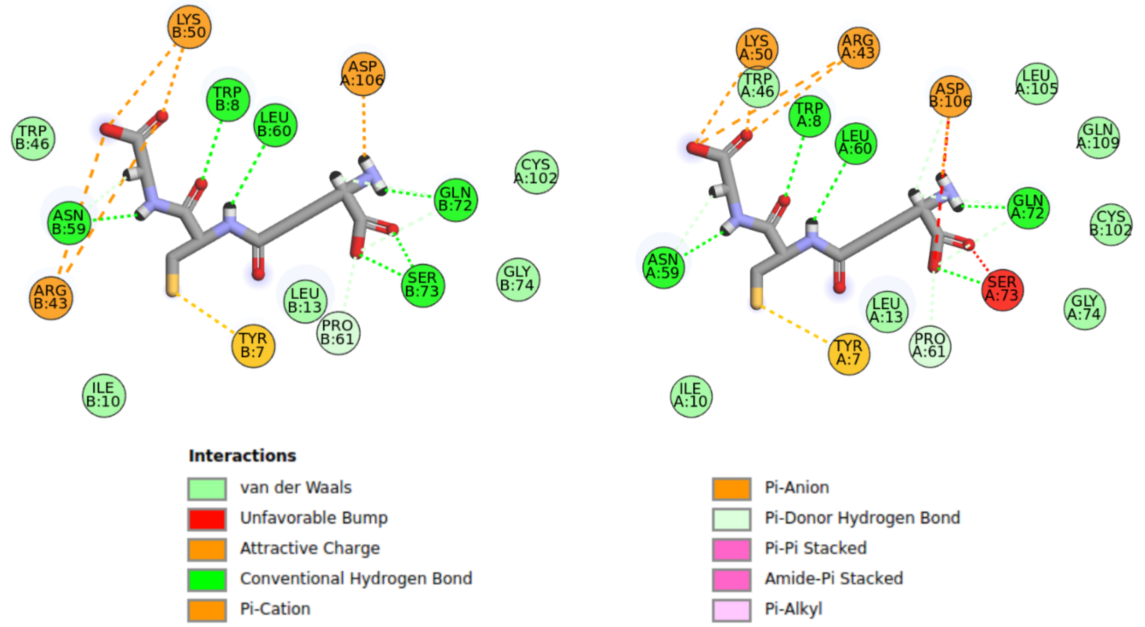

Figure S1. diagram of GSH-Ts25GST interactions identified by alignment to crystallographic structures of GST class mu complexes with glutathione.

2D interaction diagram between i11 and Ts25GST at the energy minima of the MD simulations.

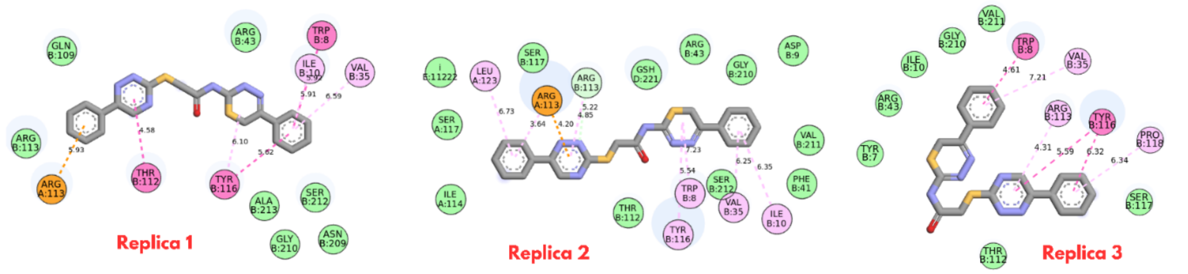

2D interaction diagram between i15 and Ts25GST at the energy minima of the MD simulations.

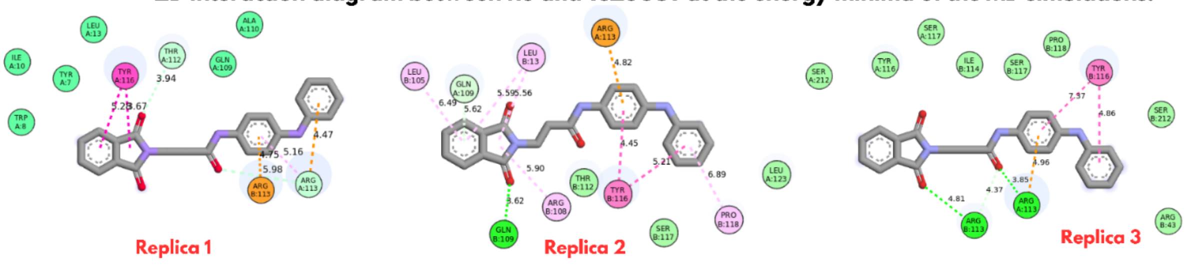

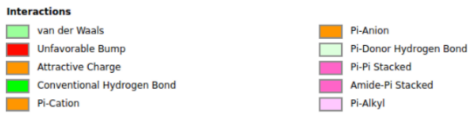

Figure S2. Results of classical calculations from Molecular Dynamics simulations of the Ts25GST-GSH complex.

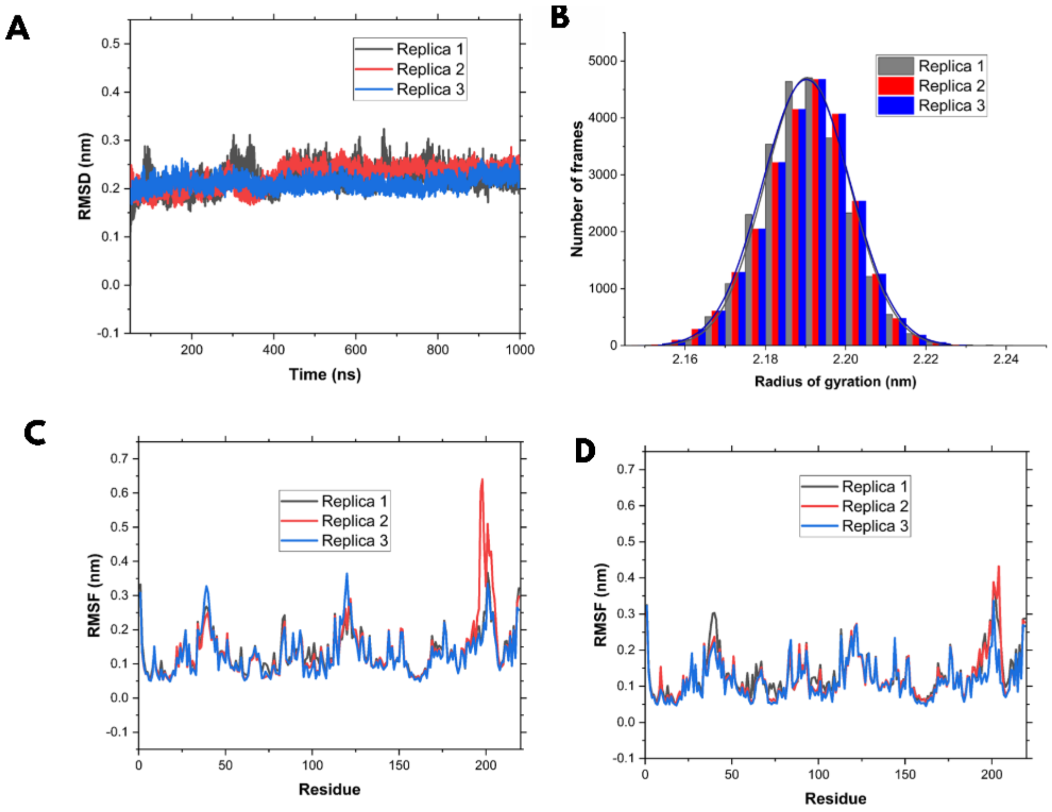

Figure S3. 2D diagrams of interaction between *i11* and *i15* with the Ts25GST-GSH complex, in the lowest energy conformations obtained from Molecular Dynamics.

Table S1. Kinetic data with variable CDNB and fixed GSH at 5 mM.

GSH 5 Mm, Ts25GST 1 ug/mL, pH 7.4 20% DMSO						
[CDNB] mM	Replica 1	Replica 2	Replica 3	Replica 4	Average	St. dev.
0.5	5.0	5.6	4.1	3.5	4.5	0.8
1	5.2	5.4	2.3	6.2	4.8	1.5
1.5	7.0	7.8	8.2	7.4	7.6	0.5
2	8.5	10.9	8.3	7.8	8.9	1.2
2.5	15.2	13.0	11.6	11.3	12.8	1.5
3	20.6	20.6	17.9	17.8	19.2	1.4
4	23.4	24.2	20.3	20.6	22.1	1.7
5	23.4	27.3	26.2	23.4	25.1	1.7
6	26.2	27.9	28.3	25.8	27.1	1.1
7	27.4	27.9	27.4	27.9	27.7	0.3
8	29.4	31.7	29.4	30.4	30.2	0.9
9	30.4	32.2	32.4	32.4	31.9	0.9
10	31.8	32.4	38.1	34.9	34.3	2.5
12	32.9	32.4	34.9	35.0	33.8	1.1

Table S2. Kinetic data with variable GSH and fixed CDNB at 7 mM.

CDNB 7 mM, 1ug/mL Ts25GST, pH 7.4 and DMSO 20%						
[GSH] mM	Replica 1	Replica 2	Replica 3	Replica 4	Average	St. dev.
0.3	9.9	10.3	9.3	10.1	9.9	0.4
0.5	12.2	11.9	13.0	12.4	12.4	0.4
0.8	12.6	13.0	12.6	12.7	12.7	0.2
1.0	14.9	15.3	14.4	14.9	14.9	0.3
1.3	17.0	17.0	17.5	17.3	17.2	0.2
1.5	19.7	20.0	19.2	19.5	19.6	0.3
2.0	22.1	21.1	21.7	21.6	21.6	0.4
2.5	23.6	22.7	23.4	23.1	23.2	0.3
3.0	26.1	24.0	27.0	25.7	25.7	1.1
4.0	28.9	25.5	29.0	27.8	27.8	1.4
5.0	29.1	26.8	33.9	29.9	29.9	2.6
6.0	31.1	31.1	32.7	31.6	31.6	0.6
7.0	31.6	32.3	32.0	32.0	32.0	0.2

No.	Library number	ID	Smiles code	Docking scores Ts25GST	
				Vina	GOLD
1	44096	7387149O1c2cc(ccc2oc1)CN1CCN(CC1)C(=O)c1cc(NC(=O)c2ccccc2)ccc1		-11.0	83.8
2	49214	7933466S1c2c(CCCC2)c(C#N)c1nc(=O)C(Oc1cc2OC(=O)C=C(c2cc1)C)C		-9.8	82.2
3	41495	6131442S1cc(nc1nc(=O)C1C2CC(CC2)C1C(O)=O)-c1ccc(cc1)C(C)C		-9.9	81.0
4	49810	7963270S1c2c(CCCC2)c(C#N)c1nc(=O)csc1nnc(n1cc)-c1ccccc1o		-9.6	85.8
5	35037	6579445Fc1ccccc1N1CCN(CC1)C(=O)C1CCN(CC1)Cc1cc(OC)ccc1		-9.9	87.4
6	46662	7779140O(c1ccc(NC(=O)coc2ccccc2)cc1)c1cc2c(cc1)C(=O)NC2=O		-10.0	81.7
7	25587	7678836S(CC(=O)Nc1c2c(ccc1)cccc2)c1oc(nn1)-c1occc1		-9.5	75.5
8	44820	7552030S(CC(=O)N1CCN(CC1)c1ccccc1)c1nnc(n1c)-c1cc(NC(=O)C)ccc1		-10.1	91.6
9	24166	7483095O1cccc1cn1c(=O)c2c(ccc(c2)C(=O)Nc2cc(ccc2)C=C)C1=O		-9.9	86.5
10	40620	5636115S1c2c(CCCC2)c(C#N)c1nc(=O)csc1nc2[nh]c3c(c2nn1)cccc3		-10.3	82.9
11	48368	7907704S1CC(=NN=C1NC(=O)csc1ncc(nn1)-c1ccccc1)c1ccccc1		-10.0	77.3
12	49330	7938228S(Cc1ccc(cc1)C(=O)Nc1ccccc1C)c1nnnn1-c1ccccc1		-10.2	88.5
13	49112	7929352Clc1ccc(-n2cnnc2)cc1c(=O)Nc1ccc(cc1)C(=O)NCC1OCCC1		-9.8	82.9
14	41277	6033159O(C)c1c(OC)cc(cc1oc)C(=O)N1CCN(CC1)C1CCN(CC1)Cc1ccccc1		-9.5	82.8
15	48083	7900497O=C1N(CCC(=O)Nc2ccc(Nc3ccccc3)cc2)C(=O)c2c1cccc2		-10.0	87.2
16	47759	7863805S1c2n=CN(CC(=O)ncc3ccccc3)C(=O)c2c(-c2cc(C)c(cc2)C)c1c		-9.8	82.8
17	48637	7913493Clc1ccc(cc1)CN(S(=O)(=O)C)CC(=O)Nc1ccccc1C(OC)=O		-9.5	89.3
18	49308	7937299Clc1ccc(-n2cnnc2)cc1c(=O)Nc1ccc(cc1)CCC(=O)N1CCCC1		-10.0	90.0
19	35032	7644562Fc1ccccc1N1CCN(CC1)C(=O)C1CCN(CC1)Cc1ccc(cc1)C		-9.8	84.8
20	45540	7644562S(CC(=O)N1CCC(N2CCCCC2)(CC1)C(=O)N)C1=Nc2c(cccc2)C(=O)N1CC		-9.5	83.8
21	44568	7504190O(c1ccc(NC(=O)CN2CCC(N3CCCCC3)(CC2)C(=O)N)cc1)c1ccccc1		-10.4	82.5
22	43451	7207711Fc1ccc(cc1)CNC(=O)c1nc(ccc1)C(=O)ncc1ccc(F)cc1		-10.0	78.3
23	47774	7865928S(=O)(=O)(ncc1ccc(cc1)C(=O)ncc1occc1)c1cc(ccc1)C(F)(F)F		-9.6	87.5
24	40233	5305148Fc1ccc(N2C(=O)C(N3CCN(CC3)Cc3cc4OCOc4cc3)CC2=O)cc1		-10.3	79.2
25	42011	6504192S(CC(=O)NC(c1ccccc1)c1ccccc1)C1=NC(O)=CC(=O)n1c1ccccc1		-10.1	88.8

26	41359	6053703O=C1N(CCCC2=Nc3c(cccc3)C(=O)N2Cc2ccccc2)C(=O)c2c1cccc2	-10.1	94.6
27	44430	7489045Clc1ccc (NC(=O)CCC)cc1nc(=O)coc1cc2c(cc1)cccc2	-9.6	84.1
28	35034	6579442Fc1ccccc1N1CCN(CC1)C(=O)C1CCN(CC1)cccc1ccccc1	-9.6	88.7

Table S3. screened Compounds

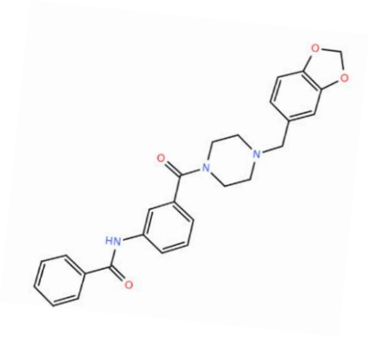

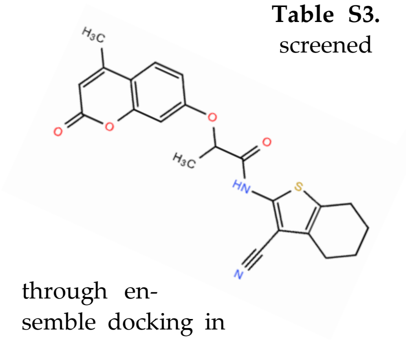

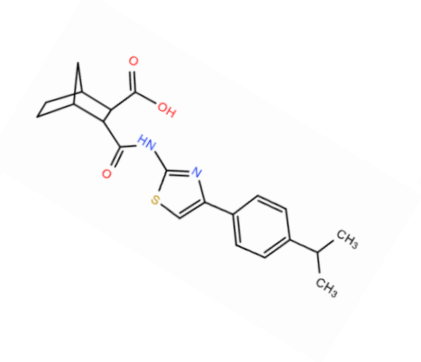

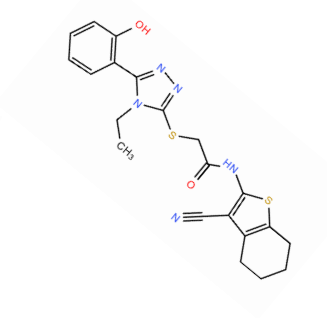

through en-
semble docking in
the discovering process.

Figure S4. 2D structures of the compounds obtained through virtual screening (part I).

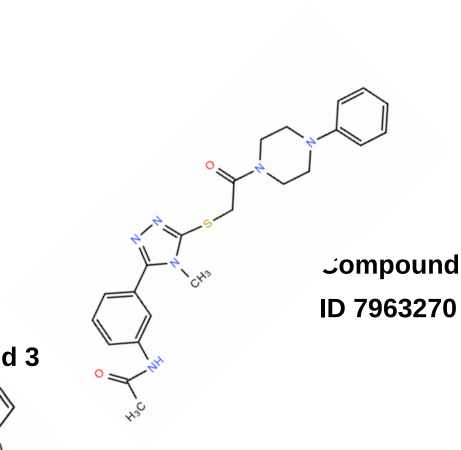

[illegible]ompound 4
ID 7963270

Co[illegible]und 1

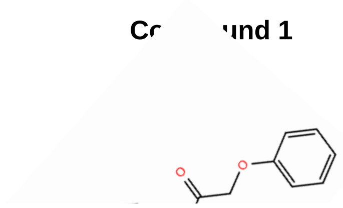

Compound 2
ID 7[illegible]

Comp[illegible]nd 3
I[illegible]

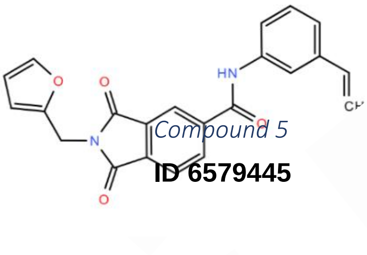

Compound 5
ID 6579445

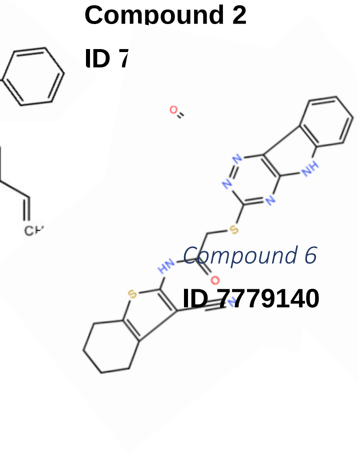

Compound 6
ID 7779140

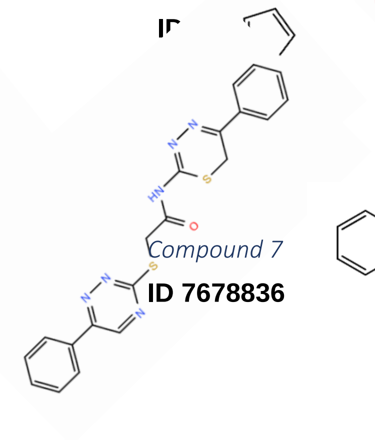

Compound 7
ID 7678836

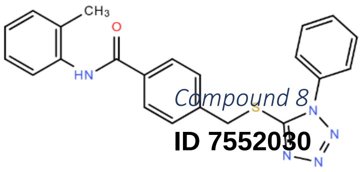

Compound 8
ID 7552030

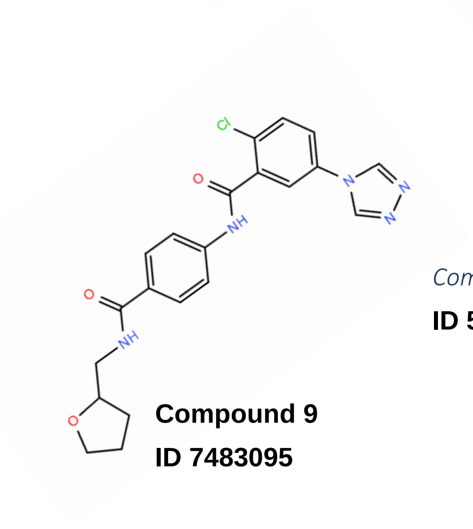

Compound 10
ID 5636115

Compound 9
ID 7483095

Compound 11
ID 7907704

Compound 12
ID 7938228

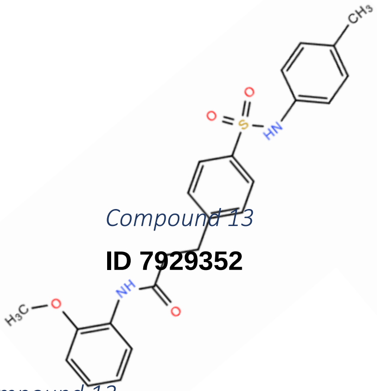

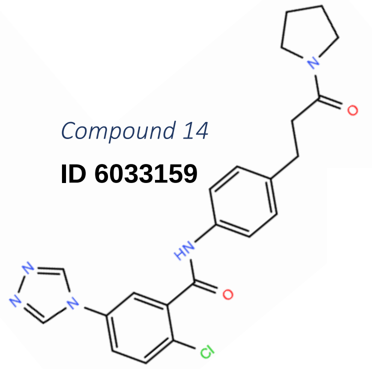

Compound 14

ID 6033159

Compound 16

ID 7863805

Compound 13

ID 7929352

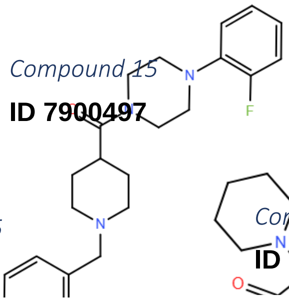

Compound 15

ID 7900497

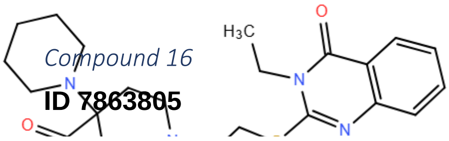

Compound 13

ID 7929352

Compound 14

ID 6033159

Compound 15

ID 7900497

Compound 16

ID 7863805

Compound 18

ID 7937299

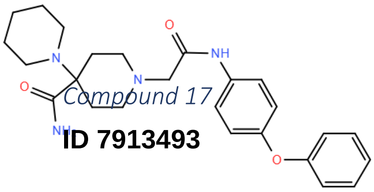

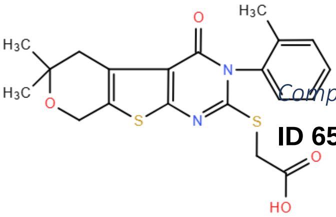

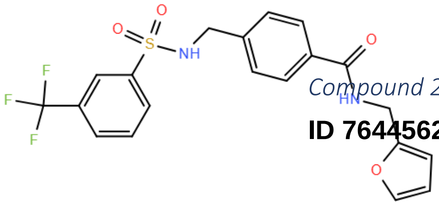

Compound 17

ID 7913493

Compound 19

ID 6579432

Compound 20

ID 7644562

Compound 21

ID 7504190

Compound 22

ID 7207711

Compound 23

ID 7865928

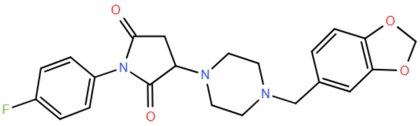

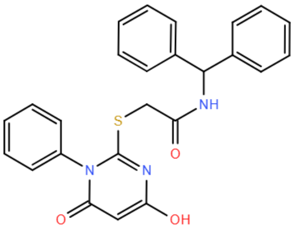

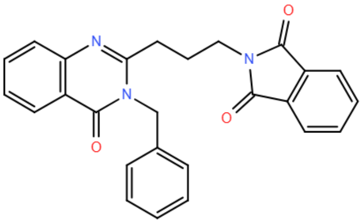

Compound 24

ID 5305148

Compound 25

ID 6504192

Compound 26

ID 6053703

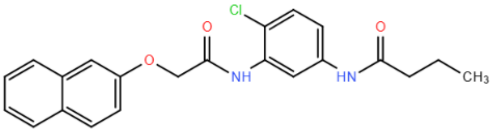

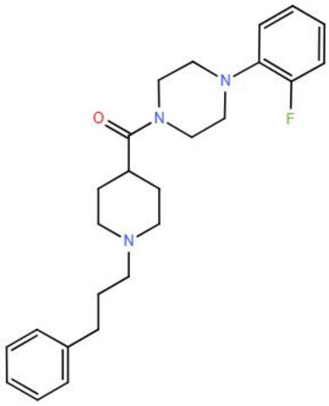

Compound 27

ID 7489045

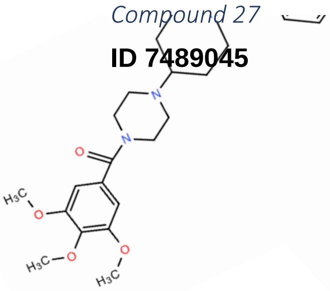

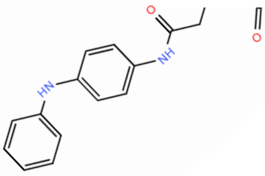

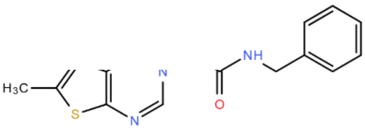

Compound 28

ID 6579442

Figure S4. 2D structures of the compounds obtained through virtual screening (part II).

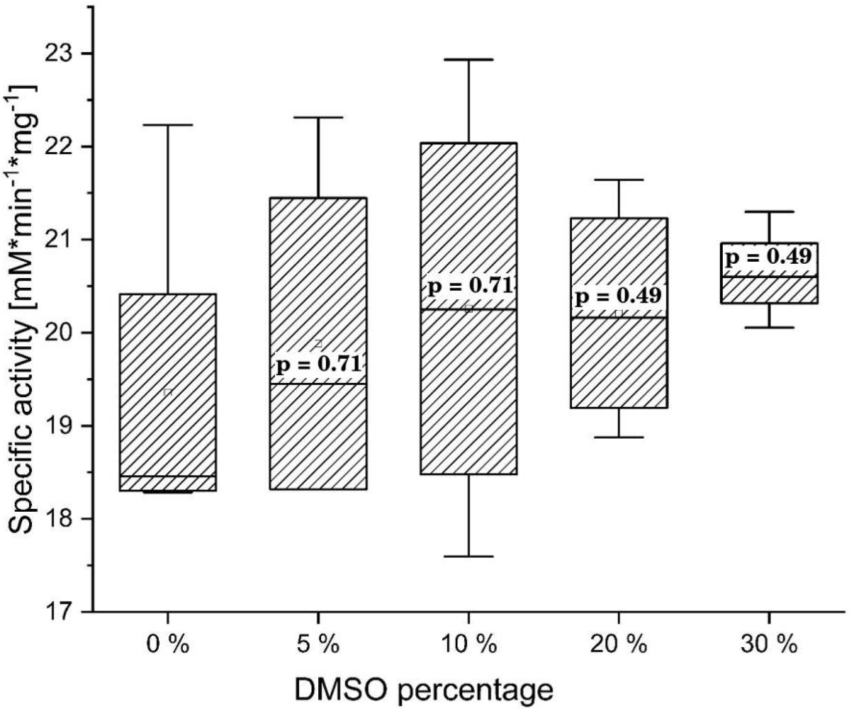

Figure S5. Results of the evaluation of the effect of DMSO on the protein activity.
